# Impaired skin microcirculation in paediatric patients with type 1 diabetes mellitus

**DOI:** 10.1186/1475-2840-12-115

**Published:** 2013-08-12

**Authors:** Mirjam Heimhalt-El Hamriti, Corinna Schreiver, Anja Noerenberg, Julia Scheffler, Ulrike Jacoby, Dieter Haffner, Dagmar-C Fischer

**Affiliations:** 1 Department of Paediatrics, University Hospital Rostock, Ernst-Heydemann-Str. 8, 18057, Rostock, Germany; 2 Department of Paediatric Kidney, Liver and Metabolic Diseases, Hannover Medical School, Hannover, Germany; 3 Present address: Department of Obstetrics and Gynaecology, University Hospital Monasterium, Münster, Germany

**Keywords:** Children, Laser Doppler Fluximetry, Skin microcirculation, Type 1 diabetes mellitus

## Abstract

**Aims/hypothesis:**

We used Laser Doppler Fluximetry (LDF) to define "normal" endothelial function in a large cohort of healthy children and adolescents and to evaluate skin microcirculation in paediatric patients with type 1 diabetes mellitus.

**Methods:**

LDF was performed in 102 healthy children (12.8 ± 3.3 years of age; 48 male) and 68 patients (12.9 ± 3.3 years of age; 33 male). Duration of disease was 5.0 ± 3.97 years. Each participant sequentially underwent three stimulation protocols (localized thermal hyperaemia with localized warming to maximum 40°C, iontophoretic delivery of pilocarpine hydrochloride (PCH) and sodium nitroprusside (SNP)). The maximum relative increase in skin blood flow and the total relative response, i.e. the area under the curve (AUC) to each stimulus (AUC_heat_, AUC_PCH_, AUC_SNP_) was determined. In addition, the area of a right-angled triangle summarizing the time to and the amplitude of the first peak, which represents the axon reflex mediated neurogenic vasodilation (ARR) was calculated.

**Results:**

In healthy controls, AUC_heat_, AUC_PCH_, AUC_SNP_, and ARR turned out to be independent of sex, age, and anthropometric values. Per parameter the 10th percentile generated from data of healthy controls was used as the lower threshold to define normal endothelial function. Diabetic patients showed significantly reduced vasodilatative response to either physical or pharmacological stimulation with SNP, whereas the response to PCH was comparable in both cohorts. In patients compared to controls *i)* a significantly higher frequency of impaired vasodilatation in response to heat and SNP was noted and *ii)* vascular response was classified as pathological in more than one of the parameters with significantly higher frequency.

**Conclusions/interpretation:**

Skin microvascular endothelial dysfunction is already present in about 25% of paediatric type 1 diabetic patients suffering from type 1 diabetes for at least one year. Future studies are needed to assess the predictive value of endothelial dysfunction in the development of long-term (cardio)vascular comorbidity in these patients.

## Introduction

Children suffering from type 1 diabetes mellitus are prone to increased cardiovascular morbidity and mortality on the long term [1-4]. Microvascular endothelial function is thought to be affected early, relative to the onset of type 1 diabetes, and an impaired endothelial function marks the beginning of the decline to clinically relevant cardiovascular disease [5,6]. An increased carotid intima-media thickness (cIMT) as a marker of macrovascular disease is seen early during the development of cardiovascular disease [7]. In contrast to cIMT, which can be measured directly and with reasonable effort, the assessment of endothelial function in-vivo is not an easy task. Several methods, e.g. laser Doppler fluximetry (LDF), laser Doppler imaging (LDI), flow mediated dilation (FMD), and videocapillaroscopy are available [8,9]. With these methods the blood flow throughout the microcirculatory network is monitored and endothelial function is assessed indirectly from the effects of exogenous manipulation of blood flow. Moreover, the anatomical localization of the microcirculatory network and the stimulus used to induce vasodilatation have to be considered [8,9]. Thus, adequate controls are required for almost every study, making the investigation of endothelial function especially in paediatric patients even more challenging. Nevertheless, several studies comparing children suffering from type 1 diabetes and age-matched healthy controls have consistently demonstrated that vascular function and cIMT are already impaired in diabetic children [2-4,6,10-15]. Apart from methodological differences for the assessment of endothelial function and despite the inherent consistency of the results reported in each of these studies the definition of "normal" endothelial function remains poor and the individual patient barely benefits from this investigations. We used LDF together with localized thermal hyperaemia as well as iontophoretic delivery of pilocarpine hydrochloride (PCH) as a stable alternative for acetylcholine chloride and sodium nitroprusside (SNP) to *i)* assess and define "normal" vasodilatory function in a large cohort of healthy children and adolescents, *ii)* compare the data from paediatric patients with type 1 diabetes to those from healthy controls in order to determine existence and frequency of cases below normal cut-off, and, *iii)* classify endothelial (dys)function in children and adolescents with type 1 diabetes and to compare the incidence between groups and factors relating to it.

### Research design and methods

#### Study design

The study received appropriate ethics committee approval from the institutional review board in accordance with the Declaration of Helsinki. Subjects and/or their parents gave written and informed consent for participating in the study.

A total of 102 *healthy* children and young adults (48 males and 54 females) with a mean age of 12.8 years (range: 6–18 years) were investigated. Participants were recruited from schools in Rostock. Paediatric patients with type 1 diabetes and a minimum age of 6 years, suffering from type 1 diabetes mellitus for at least one year and treated at our institution were invited to participate. A total of 68 patients (33 males) consented and was enrolled within a 12-month period. *Inclusion criteria:* age 6–18 years, C-peptide below 0.3 nmol/l, stable therapeutic regimen with either multiple daily insulin injections (MDII) or continuous subcutaneous insulin infusions (CSII, pump therapy) for at least 3 months. Both, healthy controls and patients were excluded in any case of febrile illness during the last three months, chronic inflammatory-/rheumatic disease, (e.g. Crohn’s disease, rheumatoid arthritis), hepatitis, HIV, glucocorticoid treatment, liver-, renal-, or cardiac failure, hereditary dyslipidaemia, skin conditions prohibiting LDF, pregnancy or tumoral diseases. All patients were free of retinal lesions and neuropathy; microalbuminuria was noted in one patient only.

## Methods

All participants were seen in our outpatient clinic in the afternoon. Demographic and clinical data were gathered by interview and chart review (i.e. mode of therapy, mean daily insulin dosages, mean HbA_1c_ during the last year), respectively. All investigations were conducted at room temperature (climatized room at 22°C) and in a quiet environment, i.e. in the absence of powerful audio-visual and other mental stimuli, and all subjects were tested individually. Participants were asked not to consume nicotine, caffeine and alcohol for at least 12 h before measurements and to take a light meal or snack at latest 2 h before the scheduled examination time. Patients do adhere to their individual therapeutic scheme. A trained physician measured weight and height throughout the study using electronic scales and a fixed stadiometer. Blood pressure (BP) was measured according to the updated Task Force Report on high blood pressure by using an oscillometric device (Dinamap 1846SX; Critikon, Tampa, USA). Calculations of individual age- and sex-related standard deviation scores (SD scores) for height, weight, BMI and BP were done as previously described [16-18]. Patients were classified as hypertensive in case of BP values above the height- and sex-related 95th percentile. Carotid intima-media-thickness (cIMT) was assessed by highresolution B-mode ultrasound using a EUB-525 Duplex Scanner (Hitachi, Tokyo/Japan) equipped with a 10-MHz linear array transducer and echo-tracking system [19]. The mean of four measurements performed in each of the two carotid arteries was used for the calculation of height-related SDS values [20,21].

### Laboratory and clinical data

In patients, the actual HbA_1c_ expressed as percentage of total haemoglobin as well as serum lipids (total cholesterol, triglycerides, LDL-cholesterol and HDL-cholesterol) and blood glucose levels were determined in the institute laboratory. The mean HbA_1c_ during the last 12 months and the mean insulin dosage per day were calculated.

### Assessment of skin microcirculation

Two laser probes, allowing simultaneous recording of skin microcirculation at different sites and conditions, a dual channel Laser Doppler Monitor (VMS-LDF2), a circular heating device with a central hole for positioning of one of the probes (SH02), a circular perspex iontophoresis chamber together with a counter electrode and the iontophoretic device (Moor Iontophoresis Controller) were used. All instruments were computer-controlled with corresponding software (MoorVMS-PC V2.0), which in turn recorded data from the laser Doppler probes with a resolution of 40 points/second. Probes were calibrated monthly and the perspex chambers were carefully rinsed with tap water and distilled water after each use. Pilocarpine hydrochloride (Caesar & Loretz GmbH, Hilden, Germany) was prepared at a final concentration of 1% (w/w) in 0.5% methylcellulose (pharmaceutical grade; Sigma-Aldrich, Taufkirchen, Germany) aliquoted and stored at 4°C until use. We used PCH as a pharmaceutically approved substitute for acetylcholine, which was barely available at the time this study was conducted. Sodium nitroprusside (SNP, analytical grade, Merck KG, Darmstadt, Germany) was freshly prepared at a final concentration of 0.1% (w/w) in 0.5% methylcellulose. In order to minimize unspecific effects, both solutions were brought to an ionic strength of 154 mmol/l by addition of sodium chloride [22].

### Protocol

Skin microcirculation was investigated essentially as described [23]. Participants acclimatized for at least 15 min and rested supine throughout the recording, which was performed in an acclimatized room (22°C). During the acclimatization period the skin at the volar surface of the forearm was gently cleaned with a skin cleanser (Octenisept, Schülke, Norderstedt, Germany). The probes used to record data at sites of baseline and stimulated blood flow were attached approximately 5 cm apart using double-sided adhesive tape (Berger Medizintechnik GmBH, Gleisdorf, Austria) avoiding hair, broken or pigmented skin, and visible blood vessels. Each participant underwent three stimulation protocols, i.e. localized heating for induction of thermal hyperaemia, iontophoretic delivery of pilocarpine hydrochloride (PCH) and sodium nitroprusside (SNP) and this sequence of events was kept constant throughout the study. The probes were repositioned after each run to avoid additive effects and/or localized exhaustion of vasodilatatory capacity.

### Thermal hyperaemia

Flow was recorded with the heating device set to 31°C for five minutes.Thereafter, the temperature was gradually increased to 40°C (increments of 2°C and finally 3°C every 2 minutes) and kept at 40°C for a further 30 minutes to ensure maximum vasodilatation [24]. In parallel, unstimulated blood flow was recorded at the control site, i.e. approximately 5 cm apart from the site of stimulation.

### Pharmacological stimulation

Endothelial-dependent and -independent vascular responses were measured using low-current iontophoresis of PCH and SNP, respectively. The iontophoresis chamber holding the laser probe in a central compartment and containing the drug solution, the counter electrode and the control laser probe were fixed 4 to 5 cm apart to the volar surface of the forearm. The iontophoresis chamber and the counter electrode were connected to the iontophoresis controller and flux was continuously recorded on both sites. In order to obtain a cumulative dose–response curve, current was applied with increasing duration and intensity (100 μA for 10 s, 1mC; 200 μA for 10 s, 2 mC; 200 μA for 20 s, 4 mC; 200 μA for 40 s, 8 mC; and 200 μA for 80 s, 16 mC) [23]. Dosages were separated by 60 s, 60 s, 90 s, and 120 s and after delivery of the last dosage the flux was recorded for an additional 180 seconds. The cumulative dosages of PCH (78.6 μg; anodal current) and SNP (95.9 μg; cathodal current) are similar at a molar base (0.3 mmol each).

### Data analysis

**Figure 1:**
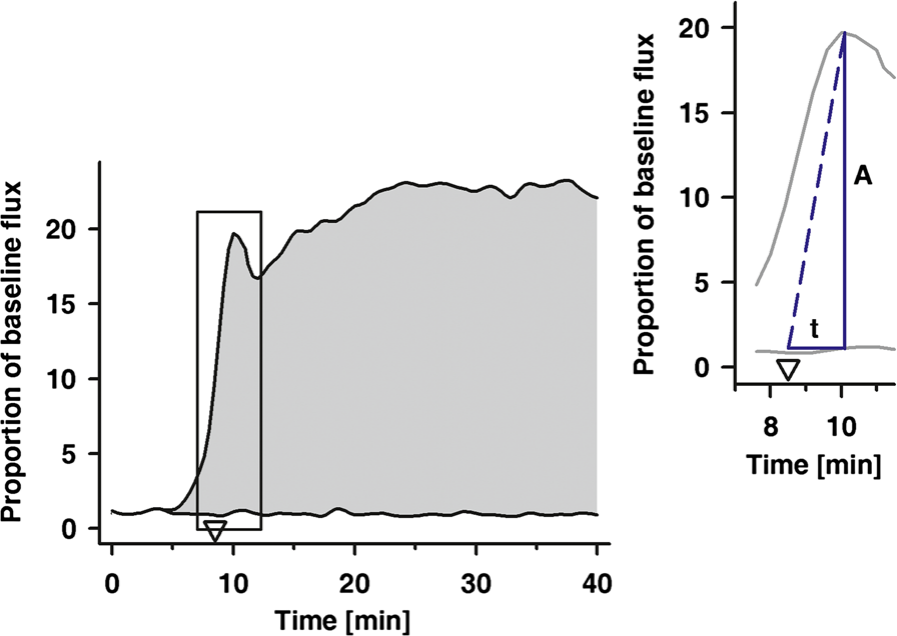
**Parameters calculated from the normalized time related response to local heat.** A, normalized axon reflex response, t, time to the axon reflex response starting from the time that the local skin temperature was set to 40°C (arrow head, 9min. after start of local warming). The area under the curve (AUC_heat_) equals the maximum response to localized heating, with beginning and end of the time interval set to 0 and 40 min, respectively. Insert: the amplitude of the axon reflex **(A)** together with the time required to peak (t) are summarized as the area of a triangle. The time interval begins 9 min after start of localized warming, i.e. the time the heating device was set to 40°C.

The mean flux measured at both sites over a period of five minutes before stimulation (baseline flux) was used for normalization of data subsequently recorded at the respective site [25-27]. In particular, the automatically recorded table containing data on time and skin blood flow (SkBF) at both sites relative to the underlying stimulation protocol were used. For either site the mean of SkBF over 5 minutes (baseline flux) was calculated and used for normalization of flux data measured at either site during stimulation. The normalized amplitude of the axon reflex response together with the normalized flux at the control site (first maximum occurring shortly after the heating device was set to 40°C) as well as the mean normalized amplitude throughout the last five minutes of localized warming (maxSkBF_heat_) were derived from these tables. For calculation of AUC_heat_, we integrated the normalized SkBF signals over the time of localized heating (i.e. 40 min) at both sites and the difference between both values is given. The two sides of the right-angled triangle, reflecting time-to and intensity of the axon reflex are the normalized amplitude and the time it took from the moment the heating device was set to 40°C (i.e. 9 min after start of the heating device) to peak (Figure 1). In the case of iontophoresis, the mean of the normalized flux signals recorded at either site for an additional period of 3min after delivery of the last drug dosage were used as maxSkBF_PCH_ and maxSkBF_SNP_, respectively. For calculation of AUC_PCH_ and AUC_SNP_ we integrated the normalized flux signals over the total time of iontophoresis, i.e. 490s of stimulation plus 180s for maximum vasodilation.

### Statistical analysis

SigmaPlot 10.0 (Systat Software GmbH, Erkrath, Germany) and SPSS 15.0 (SPSS Inc., Chicago, USA) were used for graphical presentation and statistical analysis, respectively. Normal distribution was evaluated by the Kolmogorov-Smirnow test and comparison between groups was done using Student’s t-Test or Mann–Whitney-U test, if appropriate. Nonrandom associations between categorical variables were investigated using Fisher's exact test. All p-values are two-sided and a p-value below 0.05 was considered significant. Data is given as mean ± SD or median and range, where appropriate.

## Results

**Figure 2:**
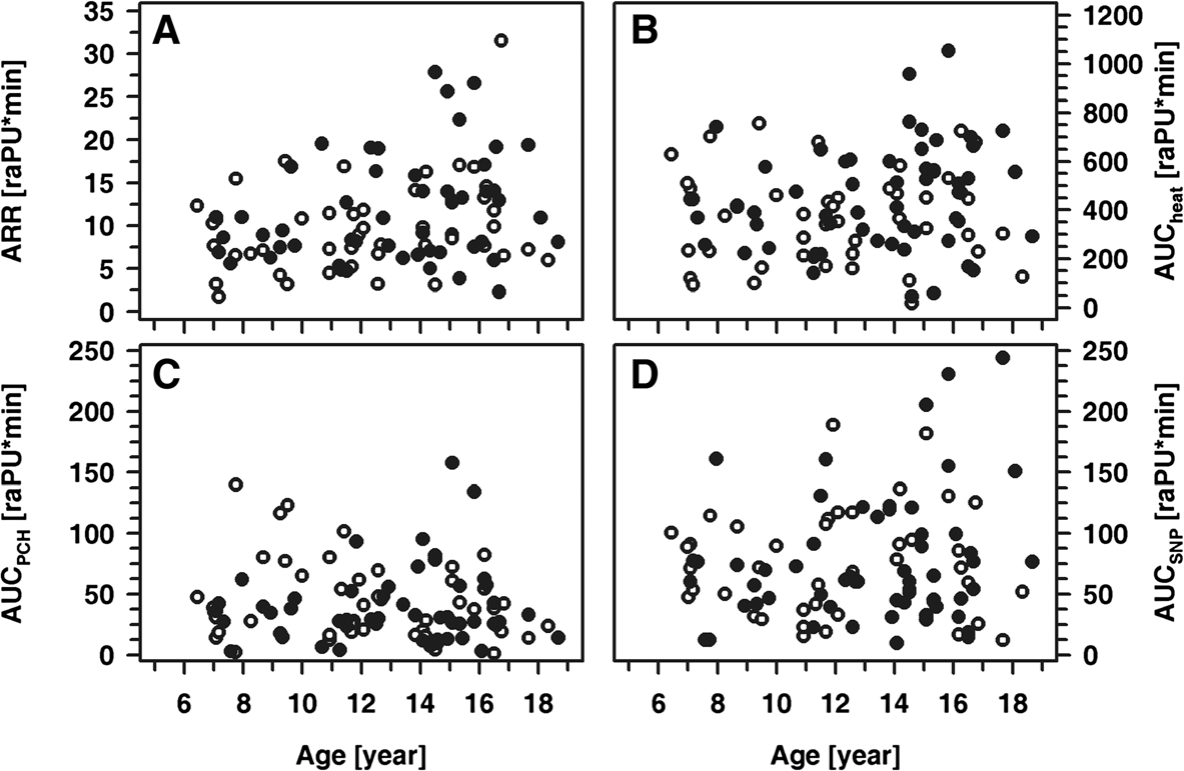
**ARR, AUC**_**heat**_**, AUC**_**PCH **_**and AUC**_**SNP **_**as a function of age in healthy children.** ARR **(A)**, AUC_heat_**(B)**, AUC_PCH_**(C)** and AUC_SNP_**(D)** as a function of age in healthy children (open symbols: male; closed symbols: female).

**Figure 3:**
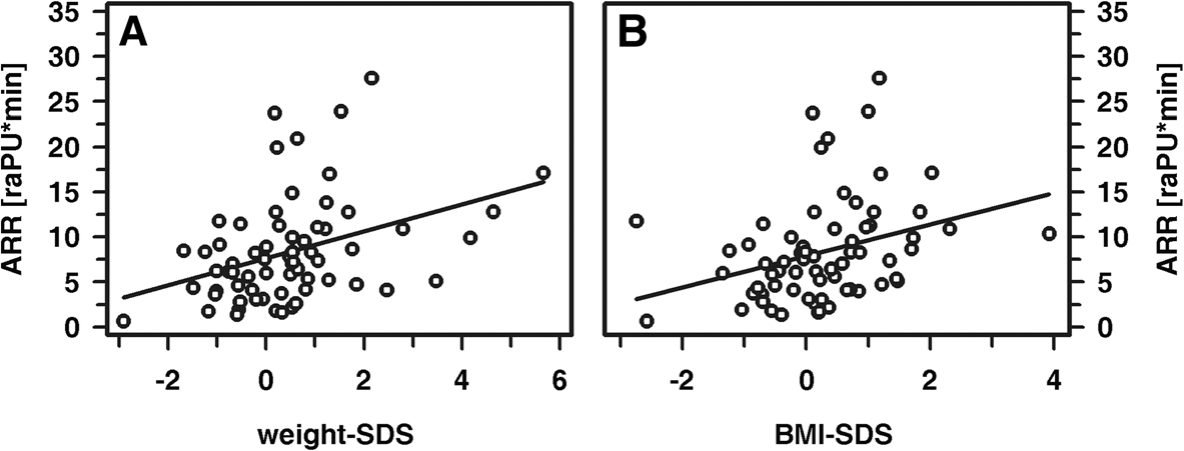
**Correlation between AAR and weight-SDS (A) and between ARR and BMI-SDS (B) in patients. A**: r= 0.37; **B**: r=0.34, each p<0.005.

**Figure 4:**
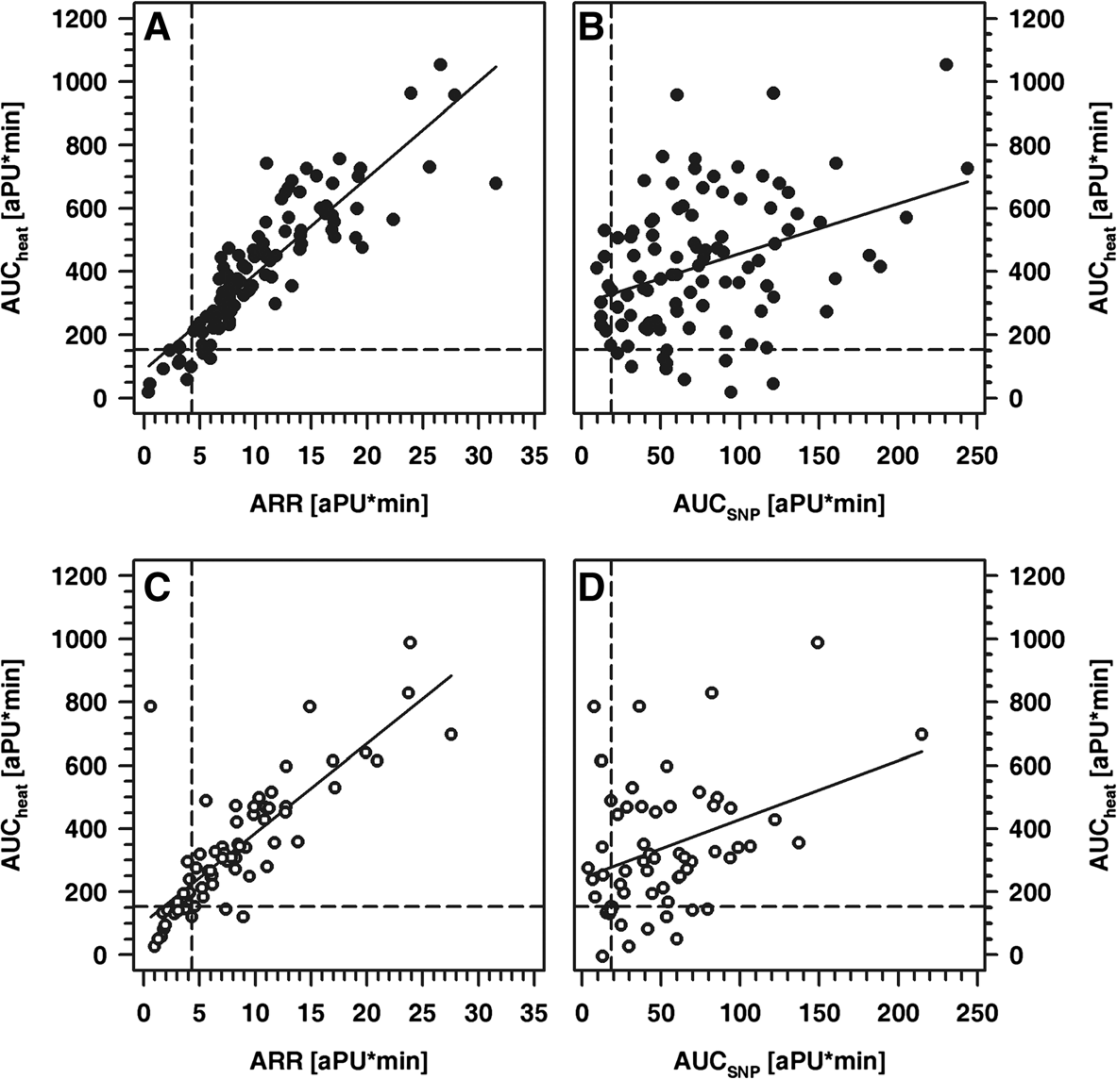
**Correlation between AUC**_**heat **_**and AAR (A, C) and between AUC**_**heat **_**and AUC**_**SNP **_**(B, D) in healthy children (A, B) and patients (C, D). A**: r= 0.92, p<0.001; **B**: r=0.37, p<0.001; **C**: r=0.80, p<0.001; **D**: r=0.32, p<0.05; per parameter the 10th percentile is indicated by a horizontal or vertical line, respectively.

Patients and healthy controls were fairly comparable with respect to age, height and weight, whereas BMI and blood pressure was significantly higher in patients compared to controls (Table 1). Interestingly, mean cIMT values did not differ significantly between patients and controls. Patients received multiple daily insulin injections (MDI; n=45) or continuous subcutaneous insulin infusions (CSII; n=23) by means of an insulin pump. All participants completed the examinations as intended and all of the procedures were well tolerated. Per participant, we had a set of four parameters, reflecting the normalized local vasodilatative capacity in response to physical (heat; AAR and AUC_heat_) and pharmacological stimulation (AUC_PCH_, AUC_SNP_). In healthy controls, these parameters were independent of sex and age as well as of absolute and standardized height, weight or BMI (Figure 2 and data not shown). Similarly, in healthy controls and patients, pubertal stage did not affect the responses to either heat, PCH or SNP. By contrast, patients' absolute and standardized weight and BMI each showed a positive correlation with ARR (ARR vs. weight: r= 0.31; ARR vs. weight-SDS: r=0.37; ARR vs. BMI: r=0.39; and ARR vs. BMI-SDS: r=0.34; each p<0.005) (Figure 3). Categorization of the patient cohort according to a normal or pathological response in either one of the parameters describing vasodilatative capacity and subsequent comparison of absolute and standardized BMI between both groups revealed no significant differences. Furthermore, in both cohorts a strong correlation between AUC_heat_ and ARR as well as a weaker one between AUC_heat_ and AUC_SNP_ was noted, while the response to PCH was apparently independent from the responses to either heat or SNP (Figure 4 and data not shown).

**Table 1:** Characteristics of patients and controls

	**Patient**	**Controls**	**p**
n	68 (33 m/35f)	102 (48 m/54f)	
Age [year]	12.93 ± 3.34	12.84 ± 3.30	0.963
	13.4 (6.3-19)	13.2 (6.4-19)	
Height			
Absolute [cm]	157 ± 18.8	158 ± 18.4	0.720
	159 (111–195)	162 (116–191)	
SDS	0.04 (−2.65-2.7)	0.37 (−2.03-2.10)	0.076
Weight			
Absolute [kg]	50.9 ± 16.3	48.0 ± 17.0	0.190
	52.6 (21.0-95.0)	48.5 (18.0-92.0)	
SDS	0.49 (−2.91-13.7)	0.17 (−0.15-3.84)	0.080
BMI			
Absolute [kg/m^2^]	19.7 (14.0-39.3)	18.2 (12.5-28.1)	0.008
SDS	0.23 (−2.74-3.92)	−0.23 (−3.20-2.51)	0.002
Systolic blood pressure			
Absolute [mm Hg]	117.1 ± 13.0	110.0 ± 11.1	<0.001
	117 (80–147)	110 (89–165)	
SDS	0.91 (−2035-2.94)	0.05 (−2.44-1.58)	<0.001
Diastolic blood pressure			
Absolute [mm Hg]	71.1 ± 9.4	65.6 ± 10.1	<0.001
	72 (39–94)	65.0 (40–105)	
SDS	0.65 (−1.65-2.35)	−0.26 (−3.23-2.0)	<0.001
cIMT [SDS]	−0.19 (3.9-3.0)	−0.31 (−2.8-1.9)	0.328
Duration of disease [year]	5.0 ± 3.97		
	4.1 (1.0-16.0)		
Blood glucose [mmol/l]	9.29 ± 4.95		
	8.60 (1.60-20.9)		
Total cholesterol [mmol/l]	4.61 ± 0.88		
	4.60 (2.6-7.3)		
LDL-cholesterol [mmol/l]	2.63 ± 0.73		
	2.66 (0.89-5.10)		
HDI-cholesterol [mmol/l]	1.54 ± 0.39		
	1.51 (0.91-2.58)		
Triglycerides [mmol/l]	1.80 ± 0.70		
	1.60 (0.60-3.66)		
Mean daily Insulin dosage [IU/kg]	093 (024–2.22)		
Actual HbA_1c_ [%]	8.65 ± 1.8	5.37 ± 0.22	<0.001
	8.3 (6.0-14.5)	5.4 (4.9-6.0)	
Mean Hba_1c_ [%]	8.62 ± 1.2		
	8.4 (6.6-11.7)		

Values are given as mean ± SD or median and range.

**Figure 5:**
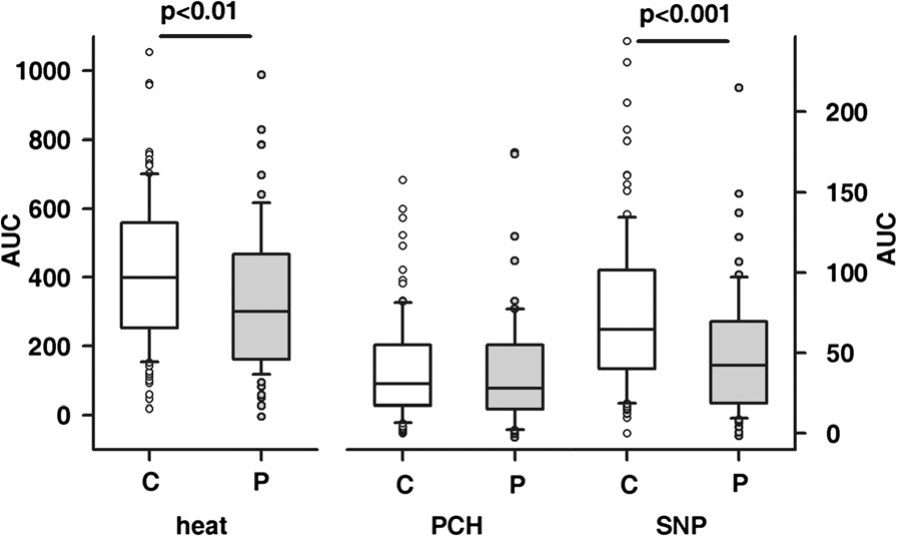
**Comparison of AUC**_**heat**_**, AUC**_**PCH **_**and AUC**_**SNP **_**in healthy controls (open bars) and diabetic patients (grey bars).**

In general, children with type 1 diabetes mellitus showed significantly reduced vasodilatative response to either physical (heat; p<0.01) or pharmacological stimulation with SNP (p<0.001) respectively, whereas the response to PCH was comparable in both cohorts (Figure 5). Given, that a low rather than high cutaneous response to induced vasodilatation reflects impairment of microvascular function, per parameter the 10th percentile was used as the lower normal cut-off, i.e. values above this threshold are considered to reflect normal vasodilatative capacity, while those below are deemed pathological. This approach confirmed that an impaired vasodilatative response with respect to stimulation with heat and SNP was significantly more frequent among children with diabetes (Table 2 and Figure 4). Interestingly, the vascular response was classified as pathological in more than one of the parameters described above with significantly higher frequency in patients compared to controls (17 out of 68 patients (25%) vs. 10 out of 102 controls (10.8%); p<0.05). In line with this, in patients with a pathological ARR also significantly lower AUC_heat_, AUC_PCH_ and AUC_SNP_ was observed (each p< 0.05). By contrast, neither cIMT-SDS nor blood pressure, duration of disease, mode of insulin therapy or serum lipids differ significantly when patients were categorized according to pathological results in either one of the four parameters defined (data not shown). However, endothelial independent vasodilatation was better preserved in patients with an insulin pump compared to those on MDI therapy (AUC_SNP_ = 64.2; 12.9 - 215 and 37.9; 1–149; CSII vs. MDI; p<0.01).

**Table 2:** Local thermal hyperaemia, SNP- and PCH-induced vasodilation in healthy controls and patients with type 1 diabetes

	**Controls**	**Patients**	**p**
ARR (cut-off: 4.3 [raPU*min])			
Median (range)	9.05 (0.40-64.9)	7.22 (0.62-27.6)	0.003
Below cut-off	10/102	18/68 (26.5%)	
AUC_heat_(cut-off:153 [raPU*min])			
Median (range)	399 (45–1053)	301 (0–988)	0.006
Below cut-off	10/102	16/68 (23.5%)	
maxSkBF_heat_(cut-off:5.9 [raPU])			
Median (range)	15.4 (1–40)	10.8 (1–36)	0.007
bBelow cut-off	10/102	12/68 (17.6%)	
AUC_SNP_ (cut-off:18.6 [raPU*min])			
Median (range)	64.6 (0–244)	41.7 (0–215)	<0.001
Below cut-off	10/102	17/68 (25%)	
maxSkBF_SNF_ (cut-off:3.0 [raPU])			
Median (range)	12.1 (1–47.8)	7.4 (1–30.9)	<0.001
Below cut-off	10/102	14/68 (20.6%)	
AUC_PCH_ (cut-off:6.6 [raPU*min])			
Median (range)	30.6 (0.158)	27.9 (0–175)	0.427
Below cut-off	10/102	8/68 (11.8%)	
maxSkBF_PCH_ (cut-off:2.1 [raPU])			
Median (range)	8.0 (1–45.6)	7.0 (1–35.4)	0.100
Below cut-off	10/102	10/68 (14.7&)	

Per parameter, median and range as well as the number of individuals with an inadequately low response to exogenous stimulation of skin microcirculation are given.

## Discussion

Type 1 diabetes is an established risk factor of cardiovascular disease, which starts from endothelial dysfunction and quietly develops over many years. The risk of cardiovascular disease depends on a variety of causes of which several can be modulated, e. g. obesity, nutritional behavior, physical activity, mode of insulin therapy, compliance with and adherence to therapeutic recommendations. The onset of type 1 diabetes is most frequently in childhood and in view of the need for a life-long therapy, compliance to therapy is a serious issue, especially during puberty. Sensitizing paediatric patients and parents to the inherent risks of severe comorbidities which become clinically visible and relevant only years after diagnosis of type 1 diabetes is challenging. Therefore, the detection of even subtle and at this stage potentially reversible nerve damage and aberrations from a normal endothelial function are of great relevance. On the one hand, this is a very logical approach to demonstrate diabetes-related complications at an early stage, on the other hand "normal" is hard to define.

Although LDF has been used to investigate microcirculation and/or the axon reflex mediated neurogenic vasodilation for more than 20 years, these issues have rarely been investigated in healthy children. Instead, LDF was used to evaluate endothelial function relative to characteristics of the disease or to compare cohorts rather than to counsel an individual patient. With LDF relative changes in skin blood flow occurring in a small volume (~1mm^3^) of tissue in response to an exogenous stimulus are accurately detected and quantified [8,26]. Due to the high sampling rate of the probes one is left with huge amounts of data reflecting the time, course and amount of vasodilatation secondary to physical or pharmacological stimulation. Instead of using several parameters describing amplitude and slope of the vascular response as a function of dose, we decided to present the individually normalized total response, i.e. for thermal hyperaemia a triangle reflecting the intensity of the axon reflex (AAR) and the areas under the curve reflecting the total effects of localized heating (AUC_heat_) or iontophoretic delivery of SNP and PCH (AUC_SNP_ and AUC_PCH_), respectively. Thus, we obtained data reflecting nerve and vascular function.

In healthy subjects none of these parameters was associated with sex, age or height. Moreover, in patients and healthy subjects pubertal stage did not affect the response to either stimulus. However, the broad and skewed distribution of the results led us to the definition of the 10th percentile as the lower threshold and discriminator between normal and impaired endothelial function in response to a given stimulus. Although this approach enables the classification of individual results and to compare subgroups of patients, i.e. those with an insufficient and those with sufficient vasodilatation none of the clinical characteristics, i.e. duration of disease, glycaemic control, serum lipids, blood pressure or cIMT, differed significantly between patients with preserved and impaired endothelial function. This might be due, at least in part, to the overall rather low vascular comorbidity in this patient cohort. In addition, more subtle measures of glycaemic control, e.g. continuous glucose monitoring, may have uncovered a significant association between glycaemic control and endothelial function in our patient cohort. None of the patients showed signs of diabetic retinopathy and only one and five out of 68 patients, respectively, presented with microalbuminuria and an increased cIMT. However, in patients with an ARR below the 10th percentile endothelial dependent and independent vasodilatation was also impaired. Thus, even we were unable to associate these findings with any of the characteristics of the underlying disease, the combination of an impaired response to either stimulus strongly indicates endothelial dysfunction. By contrast, healthy individuals usually showed such a low response for only one of the three stimuli. Although LDF might be replaced in future by more sensitive and robust laser Doppler imaging techniques, and introduction of LDF into routine clinical care is rather unlikely for several reasons, data on "normal" endothelial function in healthy children is a prerequisite for counseling of patients participating in experimental research. Previously conducted studies consistently demonstrated an impaired endothelial function in paediatric patients suffering from type 1 diabetes [2,4,10]. However, studies differ with respect to the number of patients and controls as well as to the exogenous stimulus (iontophoretic delivery of acetylcholine [10]; localized heat, iontophoretic delivery of acetylcholine and SNP [28], induction of postocclusion reactive hyperaemia [4]. Regardless of this and even of the different presentation of results, our study confirmed the expected impairment of thermal hyperaemia and NO-mediated (endothelial independent) vasodilatation in diabetic children compared to their healthy peers [2]. By contrast, in our study the response to PCH was rather low and even similar in patients and controls. First of all, this reflects the overall lower vasodilatory capacity of acetylcholine (and by analogy also PCH) compared to SNP and localized warming [29-31]. Furthermore, assessing differences between rather small absolute results of an experimental investigation, i.e. the response to PCH, requires both, highly accurate measurements with low individual variability and the examination of large numbers of individuals. Thus, the rather high variability of PCH iontophoresis and the limited number of patients may have obscured the difference between groups. Apart from this, our study and that from Khan et al. [2] are comparable with respect to *i)* the number, sex and age of patients investigated, *ii)* the duration of disease, *iii)* glycemic control, and, *iv)* daily insulin dosages, whereas the site of measurement (dorsum of the foot vs. volar surface of the forearm) and the mode of therapy (MDI and CSII vs. MDI) were different. Although categorization of our patients according to therapy revealed no statistically significant differences between patients on MDI and CSII therapy respectively, the frequency of pathological responses to heat and SNP were higher in patients on MDI therapy compared to controls, while no such difference between patients on CSII therapy and healthy controls was detectable. Whether or not this points to CSII as a more physiological mode of insulin therapy compared to MDI remains to be seen. If this holds true, one would expect lower glycaemic variability as the main contributor to endothelial and neuronal damage. Our study has some methodological limitations regarding assessment of skin blood flow. The individual variability of LDF is rather high and LDI might have been better suited for this type of study. The investigation of children required adaptation of previously established protocols, i.e. the maximum temperature for localized warming was set to 40°C instead of 42°C and blood pressure was measured only once before starting the LDF measurements. Although the stage of the menstrual cycle may have affected individual results, one could reasonably expect a similar and random distribution of this variable among both groups.

Regardless of these limitations, we could clearly demonstrate that skin microcirculation is already impaired in about 25% of paediatric patients suffering from type 1 diabetes for at least one year. Future studies are needed to assess the predictive value of endothelial dysfunction for the development of long-term (cardio-)vascular comorbidity in these patients.

## Abbreviations

ARR: Axon Reflex Response; aPU: Arbitrary perfusion units; cIMT: Carotid intima-media thickness; PCH: Pilocarpine hydrochloride; SkBF: Skin blood flow; SDS: Standard deviation score; SNP: Sodium nitroprusside.

## Competing interests

The authors declare that they have no competing interests.

## Authors’ contributions

MHE, CS, and AN performed LDF measurements and were together with JS and UJ responsible for patient care, collection and analysis of data and preparation of the manuscript. DH and DCF were responsible for the study concept and design, supervision of the study, participated in data analysis and interpretation, reviewed, edited and revised the manuscript. All authors read and approved the final manuscript.
